# *Artabotrys pachypetalus* (Annonaceae), a new species from China

**DOI:** 10.3897/phytokeys.178.64485

**Published:** 2021-05-27

**Authors:** Bine Xue, Gang-Tao Wang, Xin-Xin Zhou, Yi Huang, Yi Tong, Yongquan Li, Junhao Chen

**Affiliations:** 1 College of Horticulture and Landscape Architecture, Zhongkai University of Agriculture and Engineering, Guangzhou 510225, Guangdong, China Zhongkai University of Agriculture and Engineering Guangzhou China; 2 Hangzhou, Zhejiang, China Unaffiliated Hangzhou China; 3 Key Laboratory of Plant Resources Conservation and Sustainable Utilization, South China Botanical Garden, Chinese Academy of Sciences, Guangzhou 510650, China South China Botanical Garden, Chinese Academy of Sciences Guangzhou China; 4 Guangzhou Linfang Ecology Co., Ltd., Guangzhou, Guangdong 510520, China Guangzhou Linfang Ecology Co Guangzhou China; 5 School of Chinese Materia Medica, Guangzhou University of Chinese Medicine, Guangzhou 510006, China Guangzhou University of Chinese Medicine Guangzhou China; 6 Singapore Botanic Gardens, National Parks Board, 1 Cluny Road, 259569, Singapore Singapore Botanic Gardens Singapore Singapore

**Keywords:** Annonaceae, *
Artabotrys
*, morphology, South China, taxonomy

## Abstract

*Artabotrys pachypetalus***sp. nov.** is described from Guangdong, Guangxi, Guizhou, Hunan and Jiangxi in China. A detailed description, distribution data, along with a color plate and a line drawing are provided. In China, specimens representing this species were formerly misidentified as *A. multiflorus* or *A. hongkongensis* (= *A. blumei*). *Artabotrys blumei* typically has a single flower per inflorescence, whereas both *Artabotrys pachypetalus* and *A. multiflorus* have multiple flowers per inflorescence. In addition, *A. pachypetalus* is readily distinguished from *A. multiflorus* in having thicker and shorter petals, and connivent and somewhat trigonal or terete inner petal blades. *Artabotrys pachypetalus* is most similar to *A. punctulatus* because both have multi-flowered inflorescences and similar petal length, but *A. pachypetalus* differs in having cream petals *in vivo*, connivent inner petal blades, and a short, raised rim above the inner petal claw. *Artabotrys multiflorus* should be excluded from the flora of China because none of the Chinese specimens of *Artabotrys* collected so far fall within the variation of *A. multiflorus*.

## Introduction

*Artabotrys* R.Br. is one of the largest genera in the Annonaceae, with over 100 species of woody climbers distributed in Africa (including Madagascar) and Asia (Chen et al. 2019; Rainer and Chatrou 2021). Among the lianescent genera of the Annonaceae, *Artabotrys* is distinctive in possessing hooked inflorescence axes that facilitate climbing (Posluszny and Fisher 2000). Although the persistent inflorescence hook is a synapomorphy for the genus, identification at the species level is often complex. In general, the flowers of *Artabotrys* possess a uniform structure characterised by a tightly enclosed floral chamber (Chen et al. 2019, 2020). Both the outer and inner petals are concave at the base, with an expanded, generally flattened (sometimes terete or triquetrous) blade above the concave base. In addition, the inner petals have a projecting rim between its blade and the concave base (see fig. 9d in Chen et al. 2020); the elaborate rims of the three inner petals are tightly connivent, forming a dome around the reproductive organs. The concave base of the petals has been traditionally referred to as the ‘claw’ by Sinclair (1955) and widely followed by recent workers (e.g., Turner 2012; Prabhu et al. 2015; Turner and Utteridge 2015; Chen et al. 2018; Chen and Eiadthong 2020), although the term ‘claw’ is also used (in a strict sense) to refer to the narrowed, stalk-like, basal portion of the inner petals of other genera, e.g. *Mitrephora* (Blume) Hook.f. & Thomson, *Monodora* Dunal and *Pseuduvaria* Miq. (Sinclair 1955; Su and Saunders 2006; Couvreur 2009; Weerasooriya and Saunders 2010). The terms ‘claw’ and ‘blade’ have been explained in detail by Chen et al. (2020) in the context of the pollination biology of *Artabotrys*. To date, eight *Artabotrys* species have been recorded in China, viz. *A. fragrans* Jovet-Ast, *A. hainanensis* R.E.Fr., *A. hexapetalus* (L.f.) Bhandari, *A. blumei* Hook.f. & Thomson (recorded as *A. hongkongensis* Hance), *A. multiflorus* C.E.C. Fisch., *A. pilosus* Merr. & Chun, *A. punctulatus* C.Y.Wu ex S.H.Yuan, *A. rhynchocarpus* C.Y.Wu ex S.H.Yuan (Li and Gilbert 2011).

During our field exploration in Yangchun City in Guangdong Province, we collected an *Artabotrys* species with multi-flowered inflorescences. Morphological comparison with herbarium specimens from China revealed that the newly collected specimen is conspecific with many specimens that were previously identified as *A. multiflorus* or *A. hongkongensis*. However, our Yangchun collections (and the above-mentioned specimens) do not match type specimens or descriptions of *A. multiflorus* and *A. hongkongensis*. Originally described from Myanmar (Fischer 1937; Kress et al. 2003; Turner 2015), *Artabotrys multiflorus* also occurs in adjacent forests in Thailand (Chen et al. 2018; Chen and Eiadthong 2020) and is distinct in having multiple flowers per inflorescence and lanceolate petals (Fischer 1937; Chen and Eiadthong 2020). In China, *A. multiflorus* was first incorrectly recorded by Xu and Li (1985) based on three collections from Guizhou Province (*Z.R. Xu. L1224*, *S827*, *L437*, SYS). Subsequently, more specimens in China were misidentified as *A. multiflorus*. Thus, Li and Gilbert (2011) recorded this species in the *Flora of China* with a quite wide distribution in Guangdong, Guangxi, Guizhou and Yunnan. Originally described from Hong Kong, *A. hongkongensis* is also reportedly widespread in China, where it is known from Hunan, Guangdong, Guangxi, Yunnan, Guizhou and Hainan provinces (Li and Gilbert 2011); it is also recorded in Vietnam (Bân 2000). This species usually bears a single-flowered inflorescence (rarely two-flowered). However, this name has now been synonymized with *A. blumei* Hook.f. & Thomson (Turner 2018).

Apart from Yangchun, flowering individuals are also found in Ruyuan in Guangdong Province, as well as Mulun National Nature Reserve in Guangxi. Based on detailed comparisons of available living material as well as herbarium specimens, we confirm that our collections and many specimens misidentified as *A. multiflorus* and *A. hongkongensis* in China represent a new species described here as *Artabotrys pachypetalus*. In addition, *A. multiflorus* is not present in China, as none of the Chinese specimens of *Artabotrys* collected so far fall within the variation of *A. multiflorus*.

## Materials and methods

Physical and scanned herbarium specimens of *Artabotrys* from 15 Chinese herbaria (BNU, CCNU, GF, GFS, GZAC, GXMG, GXMI, GZTM, HGAS, HITBC, IBK, IBSC, KUN, PE and SYS; acronyms according to Thiers 2021) were studied in detail and compared with the type specimens and descriptions of *A. multiflorus*, *A. blumei* and other similar species from China and Indochina. Fresh material was collected from Chun-wan Town, Yangchun City of Guangdong, China. The morphological study was based on fresh material and herbarium specimens, but the measurements in the description below are based on dried material. The distribution, habitat and phenology of the new species are based on field observations and specimen records.

## Taxonomy

### Artabotrys pachypetalus

Taxon classificationPlantaeMagnolialesAnnonaceae

B.Xue & Junhao Chen sp. nov.

D67BF079-7997-5F32-BE25-DA53381F82E4

urn:lsid:ipni.org:names:77217250-1

Figs 1 , 2

#### Chinese name.

Hou ban ying zhua hua (厚瓣鹰爪花)

#### Diagnosis.

Similar to *Artabotrys punctulatus* C.Y.Wu ex S.H.Yuan in having multi-flowered inflorescences and similar outer and inner petal length (9–20 mm long), but differs in having non-punctate (vs. densely punctate) abaxial leaf lamina, cream (vs. beige, maroon-tinged) petals *in vivo*, connivent (vs. spreading) inner petal blades *in vivo*, a short, ca. 1 mm long (vs. an elongate, ca. 5 mm long) raised rim above the inner petal claw, and thick pericarp (2–3 mm thick vs. less than 1 mm thick).

#### Type.

China. Guangdong Province, Yangchun, Chun-wan Town, Zi-you Village, Ma-tang, alt. 110 m, limestone, 08 Apr. 2019, *X.X. Zhou, G.T. Wang* & *Y.N. Guo 0028* (holotype: IBSC [barcode no. IBSC0861926]; isotypes: KUN, SING).

**Figure 1.**
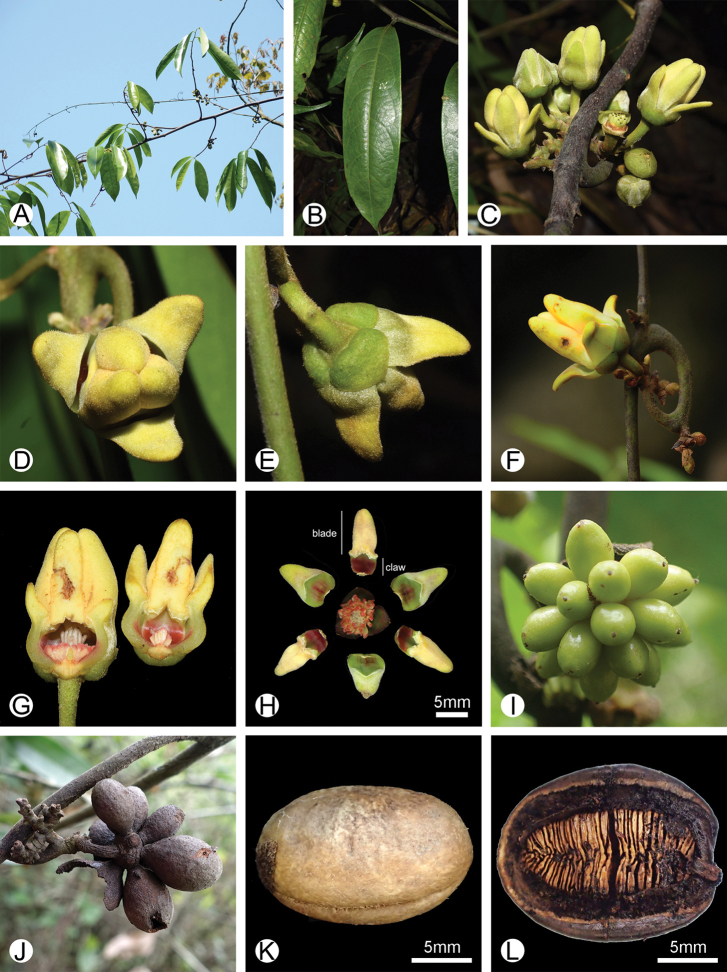
Photographs of *Artabotrys pachypetalus* sp. nov. **A** habit **B** adaxial leaf surface, showing the raised midrib **C** hooked inflorescence with many loosely clustered flowers **D** apical view of the flower **E** basal view of the flower **F** lateral view of the flower **G** longitudinal section of the flower, showing the clavate stigma and orange-red stamen connective apex **H** dissected flower, showing three sepals, three outer petals and three inner petals, and many carpels and stamens (*Y. Huang 341, IBSC*) **I** developing young fruit with many monocarps (*B. Xue XB342, IBSC*) **J** dried fruit on old branch (*B. Xue XB342, IBSC*) **K** seed (*B. Xue XB342, IBSC*) **L** longitudinal section of the seed, showing lamelliform endosperm ruminations (*B. Xue XB342, IBSC*). Photos: Yi Huang (**A, H**); Gang-Tao Wang (**B–E, G**); Yi Tong (**F**); Bine Xue (**I–L**).

#### Description.

Climbers ca. 7 m tall. Twigs drying brown, glabrous to sparsely appressed-pubescent when young. Leaf laminas chartaceous, 10–16 cm long, 3.5–5.0 cm wide, length:width ratio 3–4(–5), narrowly oblong, apex acute or acuminate to cuspidate, base cuneate, glabrous both ab- and adaxially; midrib appressed-pubescent abaxially, glabrous adaxially, raised on both surfaces; secondary veins 8–13 pairs per leaf, impressed adaxially, raised abaxially; tertiary venation reticulate, visible on both surfaces; petioles 3–5 mm long, ca. 1 mm in diameter, glabrous to sparsely appressed-pubescent, drying with transverse striations. Inflorescence axis recurved, laterally compressed, hook-like, with 6–10 flowers; flowering pedicels 6–15 mm long, ca. 1 mm in diameter, densely spreading-pubescent. Sepals 3, free, coriaceous, valvate at base, ca. 5 mm long, ca. 5 mm wide, triangular, glabrous adaxially, sparsely appressed-pubescent abaxially. Petals 6, in two whorls of 3 inner and 3 outer petals, free, valvate, coriaceous when dry, cream *in vivo*, with distinct upper blade and concave base. Outer petals 3, 9–14 mm long; blades spreading, ca. 5–9 mm long, 3–7 mm wide, ca. 1 mm thick, ovate, densely appressed-pubescent both ab- and adaxially, apex rounded; claws 4–5 mm long, 4–8 mm wide, densely appressed-pubescent abaxially, glabrous adaxially. Inner petals 3, 11–17 mm long; with a short, ca. 1 mm long raised rim above the claw; blades connivent, 7–11 mm long, 4–6 mm wide, ca. 2 mm thick, somewhat trigonal or terete *in vivo*, densely appressed-pubescent both ab- and adaxially, apex rounded; claws ca. 4–6 mm long, 3–5 mm wide, densely appressed-pubescent abaxially, glabrous adaxially, tinged orange-red at the very base *in vivo*. Stamens extrorse, ca. 50–60 per flower, ca. 2 mm long, ca. 1 mm wide; apex of connectives rounded, with a sharp edge adaxially, orange red *in vivo*. Carpels 8–20 per flower, ca. 3.5 mm long, glabrous; ovaries ellipsoid, ca. 1.5 mm long, ca. 0.6 mm wide, glabrous; ovules 2, basal; stigmas 1.5–2 mm long, cylindrical to slightly clavate, glabrous. Fruiting pedicels 10–20 mm long, ca. 3 mm in diameter, subglabrous. Monocarps 8–20 per fruit, unripe monocarps *in vivo* light green, at maturity unknown, drying black, 25–30 mm long, 15–22 mm wide, ellipsoid, glabrous, subsessile or with stipes up to ca. 2 mm long, apex rounded, pericarp 2–3 mm thick. Seeds 2 per monocarp, light yellow, smooth, plano-convex, 16–20 mm long, 12–14 mm wide, 10–12 mm thick, raphe depressed, endosperm ruminations lamelliform.

**Figure 2.**
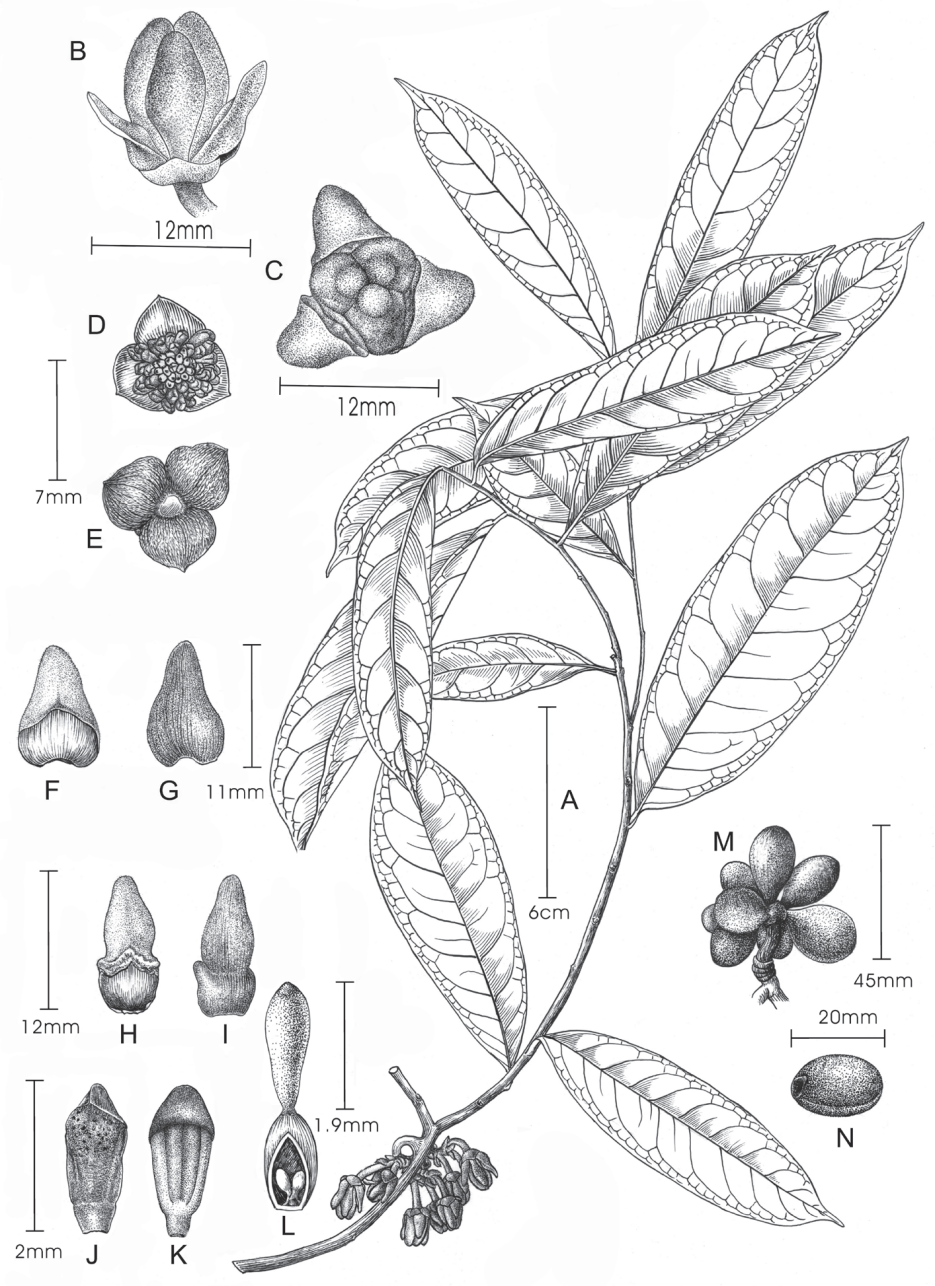
Illustration of *Artabotrys pachypetalus* sp. nov. **A** flowering branch, showing the hooked inflorescence with multiple flowers **B** lateral view of the flower **C** adaxial view of the flower, showing three outer petals and three inner petals forming a closed floral chamber **D** adaxial view of the sepals and torus with stamens and carpels **E** abaxial view of the sepals **F** adaxial view of the outer petal **G** abaxial view of the outer petal **H** adaxial view of the inner petal **I** abaxial view of the inner petal **J** adaxial view of the stamen, showing the rounded connective apex with a sharp edge **K** abaxial view of the stamen, showing four thecae **L** carpel, showing the clavate stigma and two basal ovules **M** immature fruit **N** seed. Drawn by Yun-Xiao Liu, *X.X. Zhou, G.T. Wang* & *Y.N. Guo 0028* (**A–L**); *B. Xue XB342* (**M, N**).

#### Etymology.

The specific epithet ‘*pachypetalus*’ alludes to the thick inner petals.

#### Phenology.

Flowering in April; fruiting in August to December.

#### Distribution and habitat.

**Figure 3.**
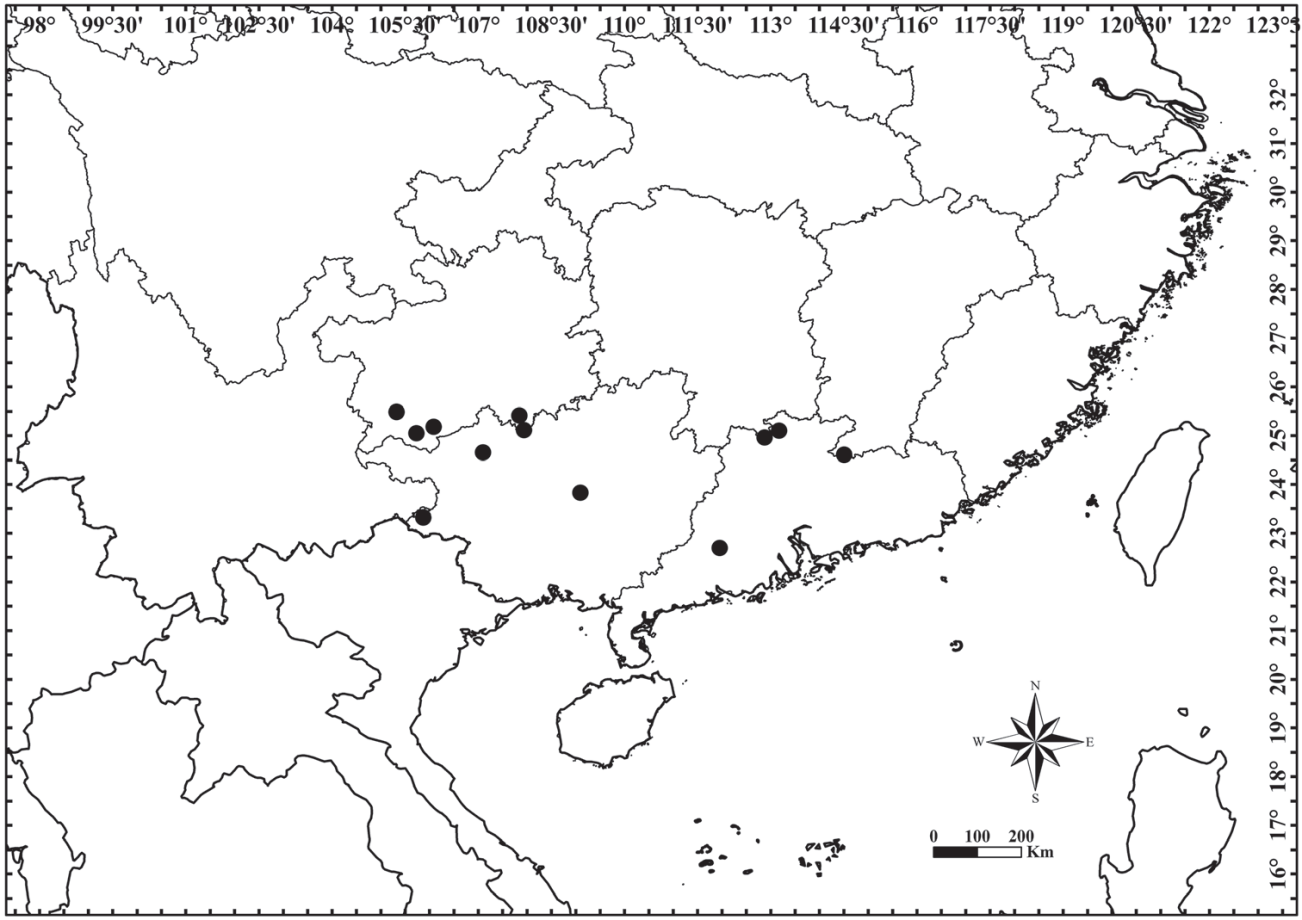
Distribution of *Artabotrys pachypetalus* sp. nov.

Guangdong, Guangxi, Guizhou, Hunan and Jiangxi (Fig. 3), at 100–1400 m elev, mainly growing on limestone.

#### Additional specimens examined

**(Paratypes).** China. **Guangdong**: Yangchun City, Chun-wan Town, Zi-you Village, Ma-tang, alt. 110 m, limestone, 13 Apr. 2020, *Y. Huang 341* (IBSC, SING); *ibid.*, 19 Apr. 2020, *B. Xue XB342, XB343* (IBSC, SING); Ru-yuan Hsien, 21 Oct. 1939, *S.K. Lau 29060* (IBSC, PE); Ru-yuan Hsien, Da-qiao Town, Da-fu village, forest valley, alt. 550 m, 13 Apr. 2013, *L. Wu & Y. Tong 3234* (BNU). **Guangxi**: Feng-shan Hsien, Qiao-yin Town, Ba-la-hou Mountain, limestone, forest edge, alt. 940 m, 3 Mar. 2013, *B.Y. Huang, X.Y. Hu & J.J. Yao 451223130331025LY* (GXMG); Qi-cheng Hisen, Cui-ping Mountain, alt. 270 m, limestone, 5 May 1983, *C.Z. Gao & A.Y. Lan 61263* (GXMI); Na-po Hisen, Nong-bu, alt. 1200 m, limestone, 02 May 1981, *Ding Fang et al. 25102* (GXMI). **Guizhou**: Li-bo Hsien, Mao-lan, 21 Apr. 1981, *J.Q. Zhang 1144* (CCNU, GF, GZAC); *ibid.*, alt. 730 m, 8 May 1981, *R.B. Jiang 81-0080* (GF, IBSC); *ibid.*, 8 May 1981, *R.B. Jiang 80* (CCNU); *ibid.*, 8 May 1981, *M.Z. Yang 810247* (HGAS); *ibid.*, alt. 750 m, 7 Mar. 1982, *Y.K. Li 10039* (IBSC); *ibid.*, alt. 710 m, 6 Sep. 1982, *Y.K. Li 10462* (HGAS); *ibid.*, alt. 800–1000 m, limestone, 03 Apr. 1984, *Z.R. Xu L1224* (IBSC); *ibid.*, 26 Apr. 1984, *K.M. Lan 840097* (GFS, GZAC); *ibid.*, 02 May 1984, *K.M. Lan 841268* (GFS, GZAC); *ibid.*, 2 May 2005, *Q.W. Sun 0505019* (GZTM); Xing-ren Hsien, alt. 1300 m, 28 Jun. 1986, *C.Z. Dang & P. Dang 156* (PE); Ce-heng Hsien, 21 May 1977, *anonymous 071*, *77-1635* (HGAS); *ibid.*, alt. 1400 m, 5 Sep. 1958, *Z.Y. Cao 0575* (PE); Wang-mo Hsien, Chengguan, forest valley, 15 May 1977, *anonymous 77-1594* (HGAS); *ibid.*, 15 May 1977, *C.Z. Dang 030* (HGAS). **Hunan**: Yi-zhang Hsien, Mang Mountain, forest valley, alt. 430 m, 16 Oct. 1942, *S.H. Chun 2490* (IBSC, IBK). **Jiangxi**: Ganzhou City, Long-nan Hsien, Jiu-lian Mountain, limestone, alt. 596 m, 05 Apr. 2021, *J.Y. Xu, L.X. Yuan, Y.R. Wang, J.R. Chen JLS-304* (SYS); *ibid.*, 09 Jun. 1970, *Group-236 0852* (PE).

## Discussion

*Artabotrys pachypetalus* is similar to *A. multiflorus* in having multiple flowers per inflorescence (Figs 1, 2), but the two species can readily be distinguished by their disparate petal morphology. The petals of *A. multiflorus* are 18–30 mm long, and 2–5 mm wide (Fischer 1937; Chen and Eiadthong 2020) whereas the petals of *A. pachypetalus* are much shorter (9–17 mm long, 3–8 mm wide). The petals of *A. pachypetalus* are also considerably thicker (1–2 mm thick when dry) than those of *A. multiflorus* (less than 1 mm thick when dry). In addition, the inner petal blades of *A. pachypetalus* are connivent and somewhat trigonal or terete whereas the inner petal blades of *A. multiflorus* are spreading and planar. Xu and Li (1985) recorded *A. multiflorus* in China based on a few collections from Li-bo Hsien, Guizhou Province (*Z.R. Xu L1224*, *S827*, *L437*, SYS). We failed to locate these specimens in SYS, but found a duplicate of *Z.R. Xu L1224* in IBSC as well as many other collections from the same locality, i.e., Mao-lan National Nature Reserve, Guizhou Province, and hence were able to confirm that they are conspecific with the new species described that we collected in Guangdong and Guangxi. The misidentification probably arose in the absence of careful comparison against the type specimens of *A. multiflorus*.

Some specimens representing *A. pachypetalus* are also misidentified with the name *A. hongkongensis* in China, which is now a synonym of *A. blumei* (Turner 2018). The distinction between these two species is quite straightforward: the inflorescence of *A. blumei* usually consists of a single flower or rarely two (Li and Gilbert 2011), whereas that of *A. pachypetalus* has 6–10 co-occurring flowers (Figs 1C, F, 2A). Additionally, *A. blumei* can be distinguished by having planar (vs. somewhat trigonal or terete), spreading (vs. connivent) inner petal blades, subglabrous (vs. sparsely to densely pubescent) pedicels and abaxial surface of sepals, and thin pericarp (< 1 mm thick vs. 2–3 mm thick).

Although *A. pachypetalus* is most similar to *A. punctulatus*, it has not been confused with the latter. This is probably because *A. punctulatus* is restricted to the montane forests of Yunnan in China and is characterized by densely punctate abaxial leaf lamina. The similarities and differences between the two species are stated in the diagnosis.

Besides the above-mentioned species, *A. pachypetalus* is also similar to *Artabotrys hienianus* Bân from Vietnam, both with multiple small hairy flowers per inflorescence (Bân 2000). The two species differ, however, in the number of the flowers per inflorescence, petiole length, leaf shape, and thickness of the pericarp. The inflorescence of *A. hienianus* has more flowers borne on shorter pedicels (5–6 mm), leading to densely clustered flowers on the inflorescence (Bân 2000), whereas that of *A. pachypetalus* has fewer flowers borne on longer pedicels (6–15 mm, Figs 1C–F, 2A), leading to loosely clustered flowers on the inflorescence (Figs 1C, 2A). *Artabotrys hienianus* has longer and broader leaves (12–17 × 5–6 cm), whereas *A. pachypetalus* has smaller and narrower leaves (10–16 × 3.5–5 cm). In addition, *A. hienianus* has a thin pericarp (Bân 2000), whereas that of *A. pachypetalus* is quite thick (2–3 mm thick) (Fig. 1L).

## Supplementary Material

XML Treatment for Artabotrys pachypetalus
